# Diet of bottlenose dolphins (*Tursiops truncatus*) from the Gulf of Cadiz: Insights from stomach content and stable isotope analyses

**DOI:** 10.1371/journal.pone.0184673

**Published:** 2017-09-12

**Authors:** Joan Giménez, Ana Marçalo, Francisco Ramírez, Philippe Verborgh, Pauline Gauffier, Ruth Esteban, Lídia Nicolau, Enrique González-Ortegón, Francisco Baldó, César Vilas, José Vingada, Manuela G. Forero, Renaud de Stephanis

**Affiliations:** 1 Departamento de Biología de la Conservación, Estación Biológica de Doñana - Consejo Superior de Investigaciones Científicas (EBD-CSIC), Isla de la Cartuja, Sevilla, Spain; 2 Centre for Environmental and Marine Studies (CESAM), Universidade de Aveiro, Campus Universitário de Santiago, Aveiro, Portugal; 3 Centro de Biologia Molecular e Ambiental (CBMA) / Sociedade Portuguesa de Vida Selvagem (SPVS), Departamento de Biologia, Universidade do Minho, Campus de Gualtar, Braga, Portugal; 4 Departament de Biologia Evolutiva, Ecologia i Ciències Ambientals, Universitat de Barcelona, Barcelona, Catalonia, Spain; 5 Conservation, Information and Research on Cetaceans (CIRCE), Algeciras-Pelayo, Cádiz, Spain; 6 Instituto de Ciencias Marinas de Andalucía - Consejo Superior de Investigaciones Científicas (ICMAN-CSIC), Campus Universitario Río San Pedro, Puerto Real, Cádiz, Spain; 7 Instituto Español de Oceanografía (IEO), Centro Oceanográfico de Cádiz, Cádiz, Spain; 8 Instituto de Investigación y Formación Agraria y Pesquera, Consejería de Agricultura, Pesca y Desarrollo Local – Junta de Andalucía – IFAPA Centro El Toruño, El Puerto de Santa María, Cádiz, Spain; University of Missouri Columbia, UNITED STATES

## Abstract

The ecological role of species can vary among populations depending on local and regional differences in diet. This is particularly true for top predators such as the bottlenose dolphin (*Tursiops truncatus*), which exhibits a highly varied diet throughout its distribution range. Local dietary assessments are therefore critical to fully understand the role of this species within marine ecosystems, as well as its interaction with important ecosystem services such as fisheries. Here, we combined stomach content analyses (SCA) and stable isotope analyses (SIA) to describe bottlenose dolphins diet in the Gulf of Cadiz (North Atlantic Ocean). Prey items identified using SCA included European conger (*Conger conger*) and European hake (*Merluccius merluccius*) as the most important ingested prey. However, mass-balance isotopic mixing model (MixSIAR), using *δ*^13^C and *δ*^15^N, indicated that the assimilated diet consisted mainly on Sparidae species (*e*.*g*. seabream, *Diplodus annularis* and *D*. *bellottii*, rubberlip grunt, *Plectorhinchus mediterraneus*, and common pandora, *Pagellus erythrinus*) and a mixture of other species including European hake, mackerels (*Scomber colias*, *S*. *japonicus* and *S*. *scombrus*), European conger, red bandfish (*Cepola macrophthalma*) and European pilchard (*Sardina pilchardus*). These contrasting results highlight differences in the temporal and taxonomic resolution of each approach, but also point to potential differences between ingested (SCA) and assimilated (SIA) diets. Both approaches provide different insights, *e*.*g*. determination of consumed fish biomass for the management of fish stocks (SCA) or identification of important assimilated prey species to the consumer (SIA).

## Introduction

Dietary information is crucial to understand the ecological role of marine top predators in an ecosystem. However, trophic information for marine mammals is difficult to obtain in the wild, as direct observations and sampling opportunities are limited by the fact that they can dive and are highly mobile [1]. Traditionally, the diet of marine mammals has been studied through stomach contents analysis (SCA) of stranded or bycaught individuals [2,3]. This technique is widely used as it provides detailed taxonomic information on diet composition [4], however it is subject to bias and limitations [5]. Differential digestion rates, degradation of identification structures, snap-shot information, uncertain representation of whole population (as information is obtained from dead animals) and undetected secondary ingestion are the main drawbacks of studying diet through SCA [5,6]. Thus, traditional techniques such as faecal or regurgitates analyses [7,8], behavioural observations [1], or molecular techniques such as stable isotopes [9], fatty acids [10] or DNA-based methods [11] are increasingly being used to complement information on stomach contents. In particular, stable isotopes analysis (SIA) has emerged as a suitable approach to reconstruct diet through mass-balance mixing models [12,13].

Dietary reconstruction based on SIA provides integrated information on the diet of predators over a longer time period than SCA [14]. Nevertheless this method is also limited by certain caveats and biases. Using stable isotopes to assess the diet of generalist and opportunistic predators can be challenging due to the broad spectrum of preys consumed [15]. Potential prey species may have similar isotopic values, thus losing taxonomic resolution when using mixing models. Therefore, coarse taxonomic estimates will be obtained when applied to generalist predators compared to the exhaustive and detailed information of SCA. Additionally, isotopic dietary reconstructions are highly sensitive to diet-to-tissue discrimination factors, which are one of the most influential parameters in the models [16].

All dietary reconstruction techniques present advantages and drawbacks [17,18]. Therefore, combining different approaches is currently considered best practice to assess the diet of top predators [1,19–21]. Additionally, these techniques are complementary because they provide information about ingested (SCA) and assimilated diet (SIA), respectively.

Bottlenose dolphin (*Tursiops truncatus*, Montagu 1821) diet has been studied in several populations worldwide [22–25], which conclude that it is a generalist predator feeding mostly on pelagic and demersal fishes [25–26]. Bottlenose dolphins are very flexible to prey on different species depending on the local availability of resources [27]. They can also display different foraging tactics where prey selection can be shaped by local ecological conditions [28]. This high variability in trophic strategies among dolphin populations requires local dietary reconstructions to consider the site-specific ecological role of the species, as well as its interaction with important ecosystem services such as marine fisheries. This is particularly important for highly-productive and overexploited marine areas such as the Gulf of Cadiz [29,30], where numerous commercial fishing fleets composed mainly of bottom-trawlers, purse-seiners and artisanal boats co-occur with bottlenose dolphins [30–32]. Furthermore, this marine food web is dominated by low trophic levels that exert an important role suggesting that possible bottom-up effects in the ecosystem are influential [29]. Nevertheless, other groups such as cephalopods and dolphins also hold an important role as top-down structuring groups [29] as seen in other locations [33]. Detailed dietary information for each cetacean species inhabiting this marine area is necessary to assess the trophic interactions among cetaceans and the top-down impact of this group on this ecosystem. In this study, we analysed the stomach content of stranded bottlenose dolphins and the relative importance of different prey types through Bayesian mass-balance mixing models (MixSIAR) in free-ranging individuals from the Gulf of Cadiz (North Atlantic Ocean).

## Materials and methods

### Stomach content analysis

Stomach contents of bottlenose dolphins were collected between 2010 and 2013 from stranded animals (n = 13). On the northern coast of the Gulf of Cadiz (37° 01’ N– 8° 59’ W / 36° 10’ N– 6° 02’ W), two stranding monitoring programs are responsible for the examination of cetacean carcasses and sample collection. On the Spanish coast, the regional government of Andalucía coordinates the program through experienced personnel and veterinarians from CEGMA (Centro de Gestión del Medio Marino Andaluz) and CREMA (Centro de Recuperación de Especies Marinas Amenazadas). On the Portuguese coast (Algarve), samples were obtained from the dedicated local stranding network, coordinated by the Portuguese Wildlife Society, under a legal licence issued by the Instituto da Conservação da Natureza e das Florestas (ICNF). The whole stomach was collected and frozen at -20°C for later examination in the laboratory. Samples were thawed and washed through different sieves (1000 μm—500 μm—300 μm) in order to separate hard parts from the remaining flesh. Cephalopod mandibles (beaks) were preserved in 70% ethanol, as were crustacean and other mollusc remains. Fish otoliths and bones were stored dry. Cephalopod beaks, fish otoliths and bones were identified using published guides [34–38] and the internal reference collection from the Portuguese Wildlife Society held in the laboratory of Ria Formosa Natural Park in Olhão (Algarve).

The number of fish was estimated from the number of otoliths (each otolith was assumed to represent 0.5 fish) or specific bones (*i*.*e*. premaxilla, maxilla, cleitrum, dentary, opercula), whichever number was higher. Otoliths were measured using a binocular microscope fitted with a digital camera to reconstruct fish length and weight. In general, otolith length was measured, except for sardine and Gobiidae otoliths, for which width is the standard measurement [36]. For otoliths identifiable to genus, family or order level, regressions based on combined data from all of the species in the group were used. For cephalopod beaks, standard measurements (rostral length for squids and hood length for octopods and sepiolids [34]) were taken on either upper or lower beaks. Dorsal mantle length (DML) and body weight of prey items were estimated using standard regressions for lower or upper beaks [34].

The relative importance of each food item in the diet in terms of presence/absence, number and estimated weight was expressed as the percentage of occurrence (%O), percentage of the total number of prey (%N) and the percentage of total prey weight (%W) [39]. The Index of Relative Importance (IRI = (%N + %W) * %O) was also computed as a summary index of dietary composition [40,41].

Confidence limits for diet composition, taking into account sampling error, were calculated by bootstrapping using the package *boot* [42] in R 2.13.0 (R Development Core Team 2008) as in Santos et al., [43]. The procedure involves the addition of all prey weights from a sample to the total diet each time a sample is selected. When *n* samples were taken, weights for each prey category were expressed as percentages of the all-categories total and the results were stored. One thousand runs were performed and the median and 95% confidence limits were calculated.

**Fig 1:**
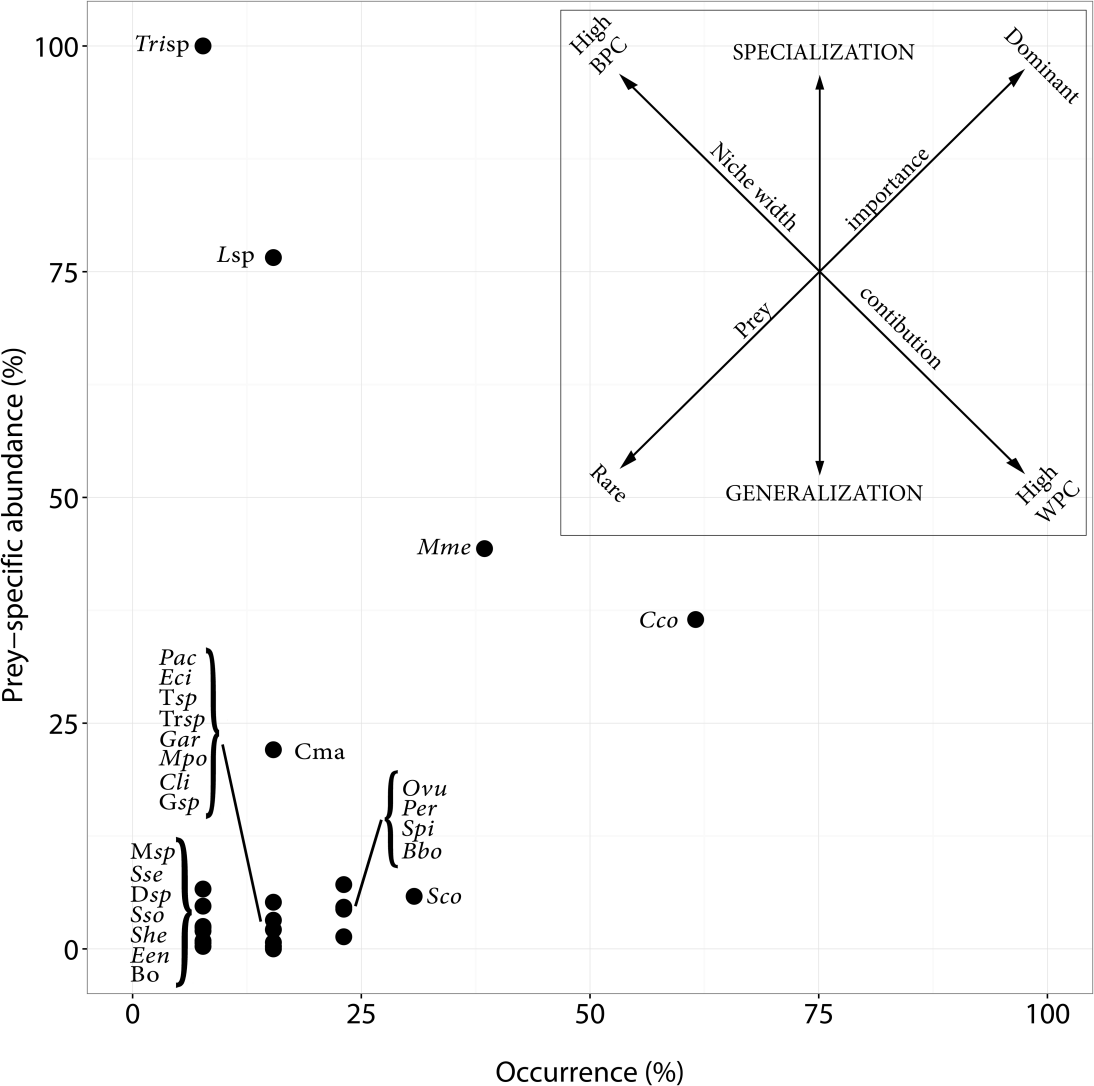
Prey-specific abundance plotted against frequency of occurrence of prey species for bottlenose dolphin from the Gulf of Cadiz. Explanatory axes for foraging patterns are those of Costello (1990) as modified from Amundsen et al. (1996). The two diagonal axes represent the importance of prey (dominant vs rare) and the contribution to the niche width (between-phenotype (BPC) vs within-phenotype contribution (WPC)); the vertical axis defines the predator feeding strategy (specialist vs generalist). ***Tri*sp**: *Trisopterus* sp.; ***Lsp***: *Liza* sp.; ***Mme***: *Merluccius merluccius*; ***Cco***: *Conger conger*; ***Cma***: *Cepola macrophthalma*; ***M*sp**: *Mugil* sp.; ***Sse***: *Solea senegalensis*; ***D*sp**: *Diplodus* sp.; ***Sso***: *Solea solea*; ***She***: *Serranus hepatus*; ***Een***: *Engraulis encraulicolus*; **Bo**: Bothidae; ***Pac***: *Pagellus acarne*; ***Eci***: *Eledone cirrhosa*; ***T*sp**: *Trachurus* sp.; ***Tr*sp**: *Trisopterus* sp.; ***Gar***: *Gadiculus argenteus*; ***Mpo***: *Micromessistius poutassou*; ***Cli***: *Citharus linguatula*; ***G*sp**: Gobidae; ***Ovu***: *Octopus vulgaris*; ***Per***: *Pagellus erythrinus*; ***Spi***: *Sardina pilchardus*; ***Bbo***: *Boops boops*; ***Sco***: *Scomber colias*.

Feeding behaviour was assessed through the construction of Costello diagram [44] modified by Amundsen et al. [45] where the percentage of occurrence (%O) was plotted against the prey-specific importance of each prey taxon (%*P*_*i*_, Eq 1):

$$\%P_{i}=\left(\frac{\sum_{i}W_{i}}{\sum_{ti}W_{ti}}\right)*100$$ (1)

where W_*i*_ is the contribution by weight of prey taxa *i* to the stomach content, W_*ti*_ is the total stomach content weight in only those predators with prey *i* in their stomachs. The position of prey types in the two-dimensional plot (Fig 1) provides information on prey importance, feeding strategy and niche width [45].

### Stable isotope analysis

Skin biopsies of free-ranging bottlenose dolphins (n = 51) were obtained via a crossbow and a modified dart with sterilised stainless-steel biopsy tips designed by Finn Larsen, following the protocols described in Giménez et al. [46] to ensure a low impact sampling method. Biopsy sampling was conducted in accordance with the ethical standards of EBD-CSIC and evaluation of its ethical committee. The project was approved and funded by the Spanish Ministry of Economy and Competitiveness [CGL2011-25543, EcoCet Project]. Fish samples were obtained from a combination of local fish markets, on-board sampling [47] and systematic sampling surveys carried out by IFAPA and the Spanish Oceanographic Institute. Immediately after collection, samples were preserved frozen at -20°C without any treatment. Dolphin skin, fish muscle and cephalopod mantle samples were dried at 60°C for 48 hours and powdered with a mortar and pestle. High lipid concentration can skew the analysis by decreasing the *δ*^13^C content [48], so lipids were removed from the samples by sequential extractions with 2:1 chloroform:methanol solution. Subsamples of powdered materials were weighed to the nearest μg and placed into tin capsules for *δ*^13^C and *δ*^15^N determinations. Isotopic analyses were carried out at the “Laboratorio de Isótopos Estables—Estación Biológica de Doñana” (LIE-EBD, Spain; www.ebd.csic.es). All samples were combusted at 1020°C using a continuous flow isotope-ratio mass spectrometry system by means of Flash HT Plus elemental analyser coupled to a Delta-V Advantage isotope ratio mass spectrometer via a CONFLO IV interface (Thermo Fisher Scientific, Bremen, Germany). The isotopic compositions are reported in the conventional delta (*δ*) per mil notation (‰), relative to Vienna Pee Dee Belemnite and atmospheric N_2_. Replicate assays of standards routinely inserted within the sampling sequence indicated analytical measurement errors of ±0.1 ‰ and ±0.2 ‰ for *δ*^13^C and *δ*^15^N, respectively. The internal standards used were: EBD-23 (cow horn), LIE-BB (whale baleen), and LIE-PA (feathers of razorbill). These laboratory standards were previously calibrated with international standards supplied by the International Atomic Energy Agency (IAEA, Vienna).

To assess the relative contributions of different prey types to the diet of the bottlenose dolphin, a Bayesian stable isotope mixing model was implemented in the MixSIAR package [49] in R 2.13.0 (R Development Core Team 2008). These models allow for the uncertainty associated with isotopic signatures and diet-to-tissue discrimination factors. A MixSIAR model was fitted with diet-to-tissue discrimination factors extracted from Giménez et al. [50], where this parameter was evaluated for the same species and tissue. The model was run with three MCMC chains, and a burn-in of 200,000 draws, followed by 300,000 draws to calculate the posterior distribution to compute credible intervals (Bayesian confidence intervals) [49]. Mass-balance mixing models provide resolved outputs when few prey species with distinct isotopic composition can be used [51]. When dealing with generalist predators that feed on a multitude of species, a reduced set of prey species or consolidating prey species is necessary due to overlapping isotopic values [19]. In this study, only important prey species detected in stomach content analysis (*i*.*e*. based on IRI values) were analysed for stable isotope determinations in order to work with a reduced dataset. A Ward’s hierarchical cluster analysis was used to group prey species in clearly separated clusters based on their mean stable isotope values. Bayesian mixing models compute prey contributions even when a model is very unlikely to satisfy the point-in-polygon assumption for every consumer (*i*.*e*. a consumer isotopic value must be within a polygon bounding the signatures of the sources [51,52]). A mixing polygon simulation was therefore constructed to determine if the mixing model design was appropriate [53]. This provided a quantitative basis for model acceptance or rejection based on a frequentist probability that the proposed mixing model can correctly calculate source contributions to explain a consumer’s isotopic value [53].

## Results

From 2010 to 2013, 13 bottlenose dolphin stomachs were analysed from the Gulf of Cadiz. In total, 1001 prey items of 35 different species belonging to 26 families were identified (Table 1). The average prey diversity in the stomachs was 6.31 species (range 1–14). Bottlenose dolphins consumed mainly fish (98.20%N, 100%O, 97.97%W, 19617 IRI), small amounts of cephalopods (1.50%N, 38.46%O, 2.03%W, 135.76 IRI) and crustaceans (0.30%N, 23.08%O). Congridae (21.48%N, 61.54%O, 35.18%W, 3486.86 IRI) was the most important family of consumed fish, followed by Merlucidae (13.69%N, 38.46%O, 16.52%W, 1161.88 IRI), Mugilidae (3.1%N, 23.08%O, 35.4%W, 888.58 IRI), Cepolidae (25.57%N, 15.38%O, 4.87%W, 468.17 IRI) and Sparidae (4.89%N, 69.23%O, 1.48%W, 440.99 IRI). Each of the main families consumed were only represented by a single species except for Mugilidae and Sparidae where two and several species were present respectively (Table 1).

**Table 1:** Diet composition of bottlenose dolphins in the Gulf of Cadiz. N = number of prey, %N = numerical percentage, O = occurrence, %O = percentage of occurrence, W = prey weight, %W = percentage of reconstructed weight, IRI = index of relative importance. 95% confidence limits are in parenthesis.

		N	%N	O	%O	W	%W	IRI
**TELEOST**								
**BOTHIDAE**		1	0.10 [0–0.54]	1	7.69 [0–23.08]	142.01	0.10 [0–0.37]	1.54 [0–21.00]
**CARANGIDAE**								
	***Trachurus* sp.**	10	1.00 [0–3.63]	2	15.38 [0–38.46]	422.50	0.29 [0–1.14]	19.84 [0–183.45]
**CENTRACANTHIDAE**								
	***Spicara maena***	2	0.20 [0–0.74]	1	7.69 [0–23.08]			
**CENTRISCIDAE**								
	***Macroramphosus* sp.**	176	17.58 [0–37.39]	1	7.69[0–23.08]			
**CEPOLIDAE**								
	***Cepola macrophthalma***	256	25.57 [0–43.77]	2	15.38 [0–38.46]	7007.84	4.87 [0–16.47]	468.17 [0–2316.83]
**CITHARIDAE**								
	***Citharus linguatula***	2	0.20 [0–0.31]	2	15.38 [0–38.46]	74.24	0.05 [0–0.18]	3.85 [0–18.85]
**CLUPEIDAE**								
	***Sardina pilchardus***	17	1.70 [0–6.88]	3	23.08 [0–46.15]	1188.95	0.83 [0–4.13]	58.39[0–508.11]
**CONGRIDAE**								
	***Conger conger***	215	21.48 [9.11–47.02]	8	61.54 [38.46–84.62]	50603.24	35.18 [19.14–64.07]	3486.86 [1086.49–9400.44]
**ENGRAULIDAE**								
	***Engraulis encrasicoliis***	1	0.10 [0–0.28]	1	7.69[0–23.08]	176.45	0.12 [0–0.48]	1.69 [0–17.54]
**GADIDAE**		24	2.34 [0.71–6.76]	7	53.9 [15.38–69.23]	249.04	0.17 [0.02–0.61]	135.29 [11.23–510.23]
	***Gadiculus argenteus***	9	0.90 [0–3.27]	2	15.38 [0–38.46]	47.93	0.03 [0–0.13]	14.30 [0–130.76]
	***Micromesistius poutassou***	4	0.40 [0–1.88]	2	15.38 [0–38.46]	15.05	0.01 [0–0.05]	6.31 [0–74.23]
	***Trisopterus* sp.**	10	1.00 [0–2.36]	2	15.38 [0–38.46]	186.06	0.13 [0–0.46]	17.38 [0–108.45]
	**unidentified Gadidae**	1	0.10 [0–0.53]	1	7.69 [0–23.08]			
**GOBIIDAE**		11	1.10 [0–3.83]	2	15.38 [0–38.46]	10.06	0.01 [0–0.02]	17.07 [0–148.07]
**HAEMULIDAE**								
	***Plectorinchus mediterraneus***	1	0.10 [0–0.49]	1	7.69 [0–23.08]			
**MERLUCIIDAE**								
	***Merluccius merluccius***	137	13.69 [3.13–35.67]	5	38.46 [15.38–61.54]	23768.36	16.52 [2.08–47.41]	1161.88 [80.18–5112.74]
**MUGILIDAE**		32	3.1 [0.05–10.68]	3	23.08 [0–46.15]	50948.96	35.4 [0–64.15]	888.58 [0–3453.40]
	***Liza* sp.**	31	3.10 [0–10.11]	2	15.38 [0–38.46]	50565.59	35.15 [0–64.24]	588.29 [0–2859.50]
	***Mugil* sp.**	1	0.10 [0–0.53]	1	7.69 [0–23.08]	383.37	0.27 [0–1.36]	2.85 [0–43.62]
**OPHIDIIDAE**								
	***Ophidion barbatum***	1	0.10 [0–0.21]	1	7.69 [0–23.08]	32.04	0.02 [0–0.11]	0.92 [0–7.39]
**SCIANIDAE**								
	***Argyrosomus regius***	3	0.30 [0–1.12]	2	15.38 [0–38.46]			
**SCOMBRIDAE**								
	***Scomber colias***	25	2.50 [0.17–8.39]	4	30.77 [7.69–61.54]	2998.07	2.08 [0.18–7.93]	140.93 [2.69–1004.33]
**SEBASTIDAE**								
	***Helicolenus dactylopterus***	2	0.20 [0–0.42]	1	7.69 [0–23.08]			
**SERRANIDAE**								
	***Serranus hepatus***	8	0.80 [0–2.39]	1	7.69 [0–23.08]	395.57	0.28 [0–1.15]	8.31 [0–81.70]
**SOLEIDAE**		3	0.3 [0–0.81]	2	15.4 [0–38.46]	744.77	0.52 [0–1.66]	12.63 [0–94.99]
	***Solea senegalensis***	1	0.10 [0–0.50]	1	7.69 [0–23.08]	274.58	0.19 [0–1.03]	2.23 [0–35.31]
	***Solea solea***	2	0.20 [0–0.63]	1	7.69 [0–23.08]	470.19	0.33 [0–1.35]	4.08[0–45.70]
**SPARIDAE**		49	4.89 [1.47–16.92]	9	69.23 [46.15–92.31]	2133.01	1.48 [0.25–6.45]	440.99 [79.38–2157.28]
	***Boops boops***	4	0.40 [0–1.36]	3	23.08 [0–46.15]	280.64	0.20 [0–0.80]	13.85 [0–99.68]
	***Dentex maroccanus***	1	0.10 [0–0.30]	1	7.69 [0–23.08]			
	***Dentex* sp.**	6	0.60 [0–2.97]	2	15.38 [0–38.46]			
	***Diplodus* sp.**	1	0.10 [0–0.48]	1	7.69 [0–23.08]	52.07	0.04 [0–0.21]	1.08 [0–15.93]
	***Pagellus acarne***	13	1.30 [0–4.19]	2	15.38 [0–38.46]	915.65	0.64 [0–2.45]	29.84 [0–255.37]
	***Pagellus erythrinus***	17	1.70 [0–6.68]	3	23.08 [0–46.15]	884.65	0.62 [0–2.76]	53.55 [0–435.66]
	***Sparus aurata***	5	0.50 [0–2.03]	2	15.38 [0–38.46]			
	**unidentified Sparidae**	2	0.20 [0–0.73]	2	15.38 [0–38.46]			
**TRICHIURIDAE**								
	***Aphanopus carbo***	1	0.10 [0–0.43]	1	7.69 [0–23.08]			
**TRIGLIDAE**		1	1.10 [0–0.40]	1	7.69 [0–20.07]			
**UNIDENTIFIED FISH**		5	0.50 [0.11–1.20]	4	30.77 [70.69–53.85]			
	**Total teleosts**	**983**	**98.20** [95.33–99.34]	**13**	**100** [100–100]	**140929.18**	**97.97** [93.82–99.82]	**19617** [18915–19916]
**CEPHALOPODS**								
**LOLIGINIDAE**								
	***Loligo vulgaris***	1	0.10 [0–0.56]	1	7.69 [0–23.08]			
**OCTOPODIDAE**		14	1.4 [0.30–3.94]	4	30.8 [7.69–61.54]	2913.03	2.03 [0.23–6.66]	
	***Octupus vulgaris***	8	0.80 [0–3.33]	3	23.08 [0–46.15]	2467.46	1.72 [0–5.63]	58.16 [0–413.50]
	***Eledone cirrhosa***	6	0.60 [0–1.23]	2	15.38 [0–38.46]	445.57	0.31 [0–1.42]	13.99 [0–101.92]
	**Total cephalopods**	**15**	**1.50** [0.37–4.50]	**5**	**38.46** [15.38–69.23]	**2913.03**	**2.03** [0.14–6.78]	**135.76** [7.84–780.91]
**CRUSTACEANS**								
**BRACHYURA**		2	0.20 [0–0.31]	2	15.38 [0–38.46]			
**ISOPODA**		1	0.10 [0–0.43]	1	7.69 [0–23.08]			
	**Total crustaceans**	**3**	**0.30** [0–0.55]	**3**	**23.08** [0–46.15]			
**TOTAL**		**1001**		**13**		**143842.21**		

Stomach content analysis of bottlenose dolphins showed a predominance of European conger (*Conger conger*) and European hake (*Merluccius merluccius*). Furthermore, cod (*Trisopterus* sp.) and mullet (*Liza* sp.) stood out in the Amundsen plot because, although they form a small occurrence, when present they are the unique or nearly unique species in the stomach (Fig 1).

**Fig 2:**
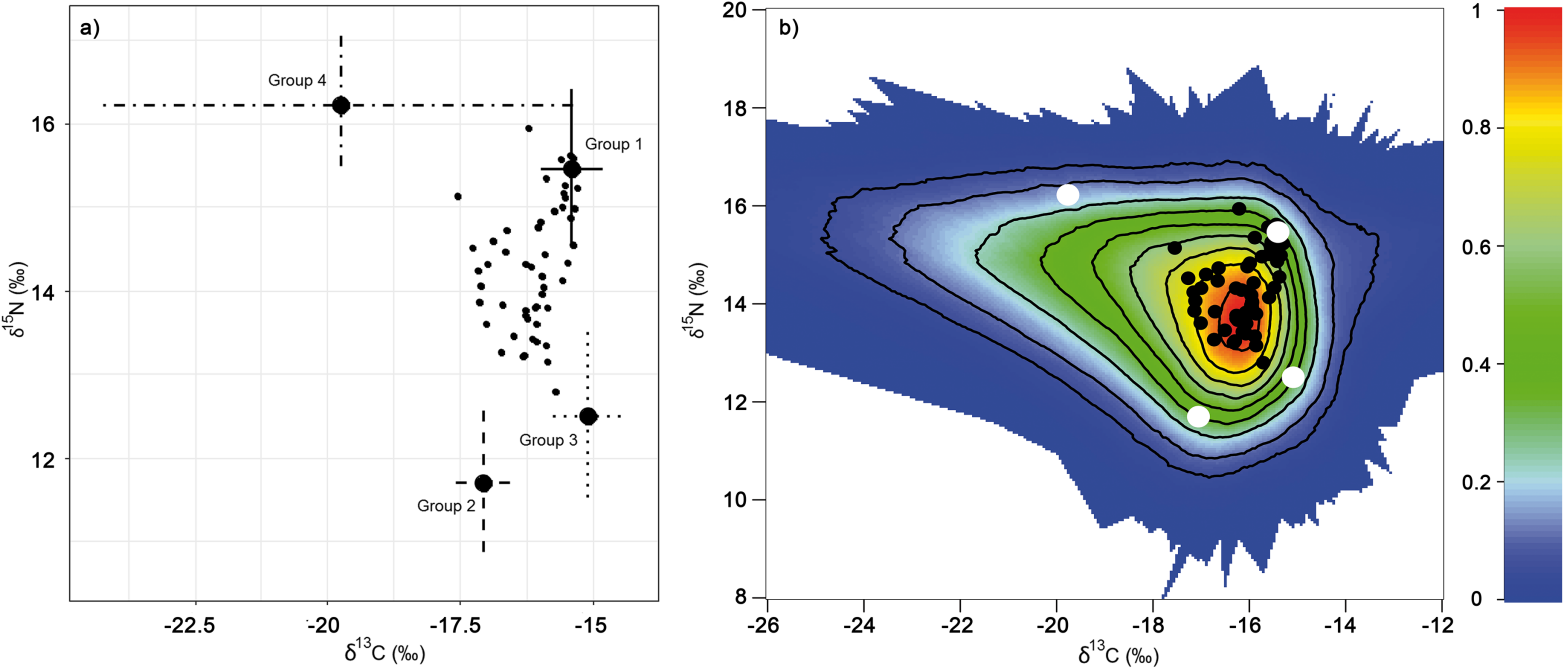
**a)** Biplot of stable isotope signatures of bottlenose dolphins (small black dots) and potential dietary sources represented with the mean value of each group and the 95% confidence intervals which incorporate the error in the source isotopic signatures and in the diet-to-tissue discrimination factors. **b)** Mixing polygon for biplot a; bottlenose dolphins are represented with black dots and potential dietary source groups with white crosses. Probability contours are drawn every 10% level. **Group 1:** *Diplodus annularis*, *Diplodus bellottii*, *Plectorhinchus mediterraneus* and *Pagellus erythrinus*; **Group 2:** *Merluccius merluccius*, *Scomber colias*, *Scomber japonicus*, *Scomber scombrus*, *Conger conger*, *Cepola macrophthalma* and *Sardina pilchardus*; **Group 3:** *Octopus vulgaris*; **Group 4:** *Liza ramada*.

**Fig 3:**
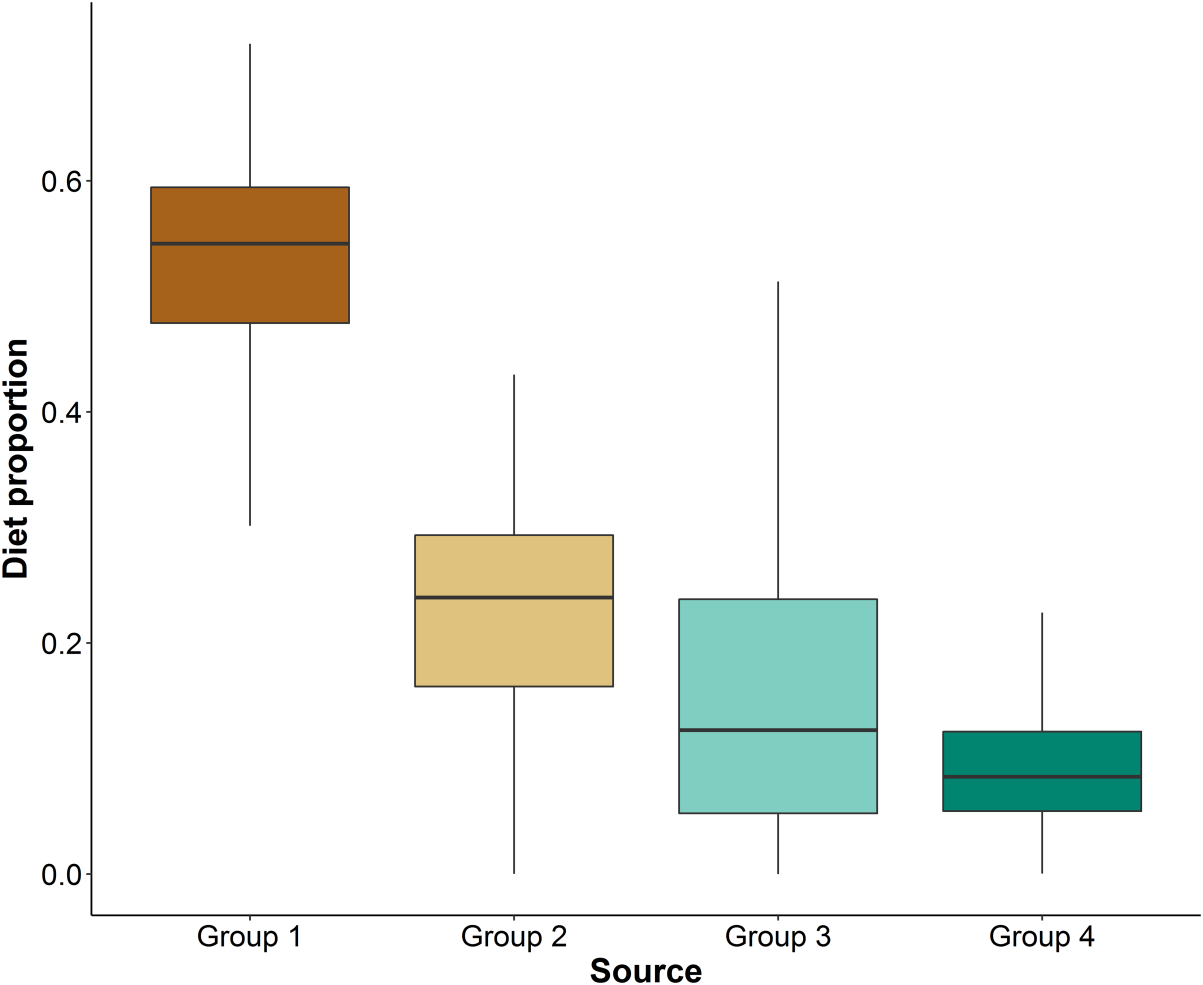
MixSIAR model results (95, 75 and 50% credibility intervals) showing estimated prey contributions to bottlenose dolphin diet in the Gulf of Cadiz. **Group 1:** *Diplodus annularis*, *Diplodus bellottii*, *Plectorhinchus mediterraneus* and *Pagellus erythrinus*; **Group 2:** *Merluccius merluccius*, *Scomber colias*, *Scomber japonicus*, *Scomber scombrus*, *Conger conger*, *Cepola macrophthalma* and *Sardina pilchardus*; **Group 3:** *Octopus vulgaris*; **Group 4:** *Liza ramada*.

Prey samples exhibited mean *δ*^13^C values ranging from -20.77 ‰ for *Liza ramada* to –15.84 ‰ for *Pagellus erythrinus* (Table 2). Regarding mean *δ*^15^N values, *Liza ramada* exhibited the highest values (15.21 ‰) and *Cepola macrophthalma* the lowest (10.05 ‰). Prey cluster analysis identified 4 well-differentiated clusters, two of them composed of only one species (Figs 2a and 3, Table 2). The mixing polygon simulation provided ground-truthing for model acceptance and validation because all the predator values fell inside the 95% mixing region (Fig 2b). The Bayesian mixing model identified group 1 and group 2 as the main contributors to bottlenose dolphin diet with 52.4% and 22.3% mean contribution respectively (Figs 2a and 3).

**Table 2:** Bottlenose dolphin and their main prey isotopic values used in the Bayesian mixing model. Group summary statistics are provided in groups where various species are included. n: number of samples, sd: standard deviation.

		*δ*^13^C			*δ*^15^N		
Species	n	mean ± sd	min	max	mean ± sd	min	max
**Bottlenose dolphins**	51	-16.13 ± 0.57	-17.55	-15.30	14.30 ± 0.76	12.80	15.94
**Group 1**	52	-16.41 ± 0.45	-17.37	-15.55	14.45 ± 0.81	12.60	15.92
*Diplodus annularis*	31	-16.58 ± 0.42	-17.37	-15.56	14.43 ± 0.85	12.60	15.92
*Diplodus bellottii*	9	-16.42 ± 0.22	-16.73	-16.05	15.14 ± 0.20	14.74	15.37
*Plectorhinchus mediterraneus*	2	-16.64 ± 0.23	-16.80	-16.47	15.00 ± 0.04	14.97	15.02
*Pagellus erythrinus*	10	-15.84 ± 0.25	-16.38	-15.55	13.79 ± 0.50	13.24	14.73
**Group 2**	120	-18.07 ± 0.67	-19.64	-16.56	10.69 ± 0.96	8.36	13.21
*Merluccius merluccius*	31	-18.23 ± 0.66	-19.44	-16.56	10.86 ± 0.89	9.66	13.21
*Scomber colias*	20	-18.41 ± 0.43	-19.25	-17.63	10.99 ± 0.45	10.30	11.74
*Scomber scombrus*	10	-18.26 ± 0.18	-18.47	-18.02	11.13 ± 0.57	10.31	12.04
*Cepola macrophthalma*	9	-17.53 ± 0.27	-18.02	-17.17	10.05 ± 0.40	9.50	10.69
*Conger conger*	10	-17.26 ± 0.18	-17.56	-17.00	10.91 ± 0.33	10.15	11.32
*Sardina pilchardus*	40	-18.04 ± 0.77	-19.64	-16.99	10.38 ± 1.27	8.36	13.07
**Group 3**							
*Octopus vulgaris*	11	-16.10 ± 0.73	-16.96	-14.36	11.49 ± 0.98	10.02	13.14
**Group 4**							
*Liza ramada*	5	-20.77 ± 4.58	-27.15	-15.28	15.21 ± 0.71	14.00	15.79

## Discussion

Diet analyses of marine top predators are essential to understand the structure and behaviour of marine communities, as they have been recognized as keystone species worldwide [54]. In the Gulf of Cadiz, SCA demonstrate that bottlenose dolphins primarily consume European conger and European hake, although 35 different fish and invertebrate species were detected in the stomachs of stranded animals. Therefore, bottlenose dolphins can be considered generalist predators in this area. On the other hand, SIA highlighted that the most assimilated prey items were Sparidae species.

Overall, the results obtained in the present study are similar to the studies performed elsewhere around Europe, where bottlenose dolphin diet comprises primarily demersal and some pelagic fishes [2,25,55,56]. For instance, the European hake has also been identified as one of the main prey species of bottlenose dolphin populations in the waters surrounding France and Galicia (North-west Spain) and in the western Mediterranean Sea [25,27,55]. The consumption of conger eels has also been reported for other European populations, although their contribution highly varies among localities [25,27,55,56]. In contrast, these studies demonstrated that Sparidae species are secondary prey species. The importance of Mugilidae in the present study may be due to the presence of bottlenose dolphins in coastal waters and some incursions into the Odiel and Guadalquivir rivers (Carlos Gutiérrez-Expósito and Francisco Baldó personal communication) where the range of both species overlap. Differences in bottlenose dolphin feeding ecology in different areas may have been produced by local adaptation to different habitats with diverse ecological opportunities [57].

Neither SCA nor SIA provide a perfect estimation of true predator diet, therefore the use of both techniques is desirable to overcome aforementioned caveats. Each technique provides different information; SCA provides information on the ingested diet while SIA reveals the assimilated diet. Thus, a multi-technique approach allows assessing if feeding preferences are consistent across multiple time-scales. The integration time (*i*.*e*. information window provided) of each technique is different, with a longer integration time for stable isotopes. In addition, dissimilar results may arise due to different assimilation efficiencies between species consumed. Therefore, depending on the research question being posed, one could choose one or the other technique, but the combination of both techniques provides a more complete understanding of the role of this predator in the ecosystem. Stomach content analysis may be more useful to assess the overlap and competition with local fisheries or the impact of this predator on ecosystem functioning, as we can assess the biomass removed by the predator with high taxonomic precision [15,58]. However, if the focus is on metabolism and energetics, then SIA is preferable to SCA as it pertains to the assimilated diet [59]. In addition, other techniques such as fatty acids analysis could have been used to enhance the taxonomic resolution of assimilated diet assessment.

Recently, Santos et al., [60] quantified the cetacean predation on sardine and European hake in the Atlantic waters of the Iberian Peninsula. However, they were forced to extrapolate bottlenose dolphin diet information obtained from the northern Iberian Peninsula to the Gulf of Cadiz. Consequently, this extrapolation in conjunction with other data limitations (*i*.*e*. energy requirements and population estimates) may have caused unrealistic estimates of predation exceeding the estimated hake natural mortality [60]. Nevertheless, it seems that bottlenose dolphins may play an important role in determining hake stock dynamics [60]. Models from Santos et al. [60] could integrate the new information about southern Iberian dolphins from the present study to assess the actual impact of bottlenose dolphins on hake population dynamics. Additionally, multi-species mass-balance models (*i*.*e*. Ecopath) performed by Torres et al., [29] in the Gulf of Cadiz should also be updated. We should move towards modelling small cetacean species present in the Gulf of Cadiz as individual functional groups [61] instead of grouping them in a single group (*i*.*e*. dolphins functional group), because different cetacean species may present quite different diets. Therefore, more realistic models can be obtained and we could accurately assess the trophic links of different cetacean species in this highly impacted ecosystem.

The high fishing pressure in the Gulf of Cadiz [30,31,62] may induce ecosystem changes altering the present food web structure. Marine mammals have been proposed as an ecological indicator to monitor fishing impacts [54]. Additionally, bottlenose dolphins are one of the functional groups in the European Marine Strategy Framework directive (MSFD, 2008/56/EC), classified as “ecologically relevant” and therefore must be monitored to achieve a good environmental status by 2020 [63].

This study provided local dietary information for this dolphin population. Based on our results, we recommend monitoring temporal changes in the bottlenose dolphin diet to detect ecosystem changes in this highly fishery exploited area. Moreover, understanding the dynamic processes of trophic interactions will help to determine the impact of anthropogenic changes in this marine ecosystem.
